# Hypoxia–ROS–HSP Triad Axis in Cancer: Mechanisms, Crosstalk, and Therapeutic Implications

**DOI:** 10.3390/cells15191802

**Published:** 2026-10-02

**Authors:** Manish Kumar Singh, Hyeong Rok Yun, Jyotsna Ranbhise, Sunhee Han, Seung Geun Yeo, Fu-Shi Quan, Bonglee Kim, Sung Soo Kim, Insug Kang

**Affiliations:** 1 Department of Biochemistry and Molecular Biology, School of Medicine, Kyung Hee University, Seoul 02447, Republic of Korea; manishkhu05@khu.ac.kr (M.K.S.);; 2 Biomedical Science Institute, Kyung Hee University, Seoul 02447, Republic of Korea; 3 Department of Biomedical Science, Graduate School, Kyung Hee University, Seoul 02447, Republic of Korea; 4 Department of Otorhinolaryngology—Head and Neck Surgery, College of Medicine, Kyung Hee University Medical Center, Kyung Hee University, Seoul 02453, Republic of Korea; 5 Department of Medical Zoology, Medical Research Center for Bioreaction to Reactive Oxygen Species and Biomedical Science Institute, Core Research Institute, School of Medicine, Kyung Hee University, Seoul 02447, Republic of Korea; 6 Department of Pathology, College of Korean Medicine, Kyung Hee University, Seoul 02447, Republic of Korea

**Keywords:** cancer, hypoxia, oxidative stress, ferroptosis, heat shock proteins, mitochondria, cellular homeostasis

## Abstract

**Highlights:**

**What are the main findings?**

- This review highlights how three critical processes, namely, hypoxia, ROS, and heat shock proteins (HSPs), interplay in cancer establishment and survival.
- We demonstrate how diverse upstream stimuli and stressors within the tumor microenvironment converge upon common, progression-related signaling circuits through the synergistic interaction of this specific Hypoxia-ROS-HSP triad.

**What are the implications of the main findings?**

- A central focus is the dual functionality of HSPs as conventional molecular chaperones, acting as essential survival factors for cancer cells under stress, while simultaneously presenting as vulnerabilities that can be therapeutically exploited.
- By establishing the critical role of this triad axis in driving therapy resistance, these findings position the Hypoxia-ROS-HSP axis as a high-priority, actionable target for the development of novel therapeutic strategies to overcome treatment-refractory cancers.

**Abstract:**

Cancer remains one of the leading causes of mortality worldwide, accounting for approximately 20% of all deaths and posing a major threat to global health. Mechanistically, tumorigenesis is driven by complex molecular and cellular mechanisms that enable malignant cells to evade current therapeutic interventions, thereby limiting treatment efficacy and contributing to disease progression. Despite extensive efforts to identify and target key molecular regulators and signaling pathways involved in cancer development, effective and durable treatments remain limited for many cancers, underscoring the need for novel treatment strategies. Consequently, diverse therapeutic strategies are being developed to overcome barriers to treatment response, improve clinical outcomes, and extend patient survival. Among emerging therapeutic targets, HSPs have attracted considerable attention because of their critical roles in maintaining proteostasis, promoting tumor cell survival, and facilitating resistance to therapy. In addition to stress adaptation, oxidative stress and hypoxia are key components of the tumor microenvironment that promote malignant progression through extensive crosstalk with oncogenic signaling pathways. Growing evidence indicates that these pathways are highly interconnected and collectively contribute to cancer metastasis and poor clinical outcomes. In this review, we explore the mechanistic interplay among HSPs, reactive oxygen species (ROS), and hypoxia across various cancers, highlight their potential as integrated therapeutic targets, and explore future strategies to develop more effective anticancer interventions.

## 1. Introduction

Cancer is a multifactorial disease characterized by the uncontrolled proliferation of malignant cells that invade surrounding tissues and disseminate to distant organs via metastasis. The aggressiveness of cancer is reflected by its stage of progression and metastasis potential. Numerous risk factors contribute to cancer initiation and progression, including aging, obesity, unhealthy dietary habits, excessive consumption of alcohol and tobacco, environmental pollution, genomic instability, and sedentary lifestyle. Despite significant advances in diagnosis and treatment, cancer remains a major global health challenge, particularly when detected at advanced stages, where metastasis substantially reduces patient survival rates [1]. According to the World Health Organization (WHO), cancer is one of the leading causes of mortality worldwide, accounting for nearly 10 million deaths in 2022 [2]. Cancer progression is driven by a complex interplay of genetic, epigenetic, and microenvironmental factors. Among these, oxidative stress, hypoxia, and HSPs have emerged as critical regulators of tumor initiation, growth, metastasis, and therapy resistance.

**Figure 1:**
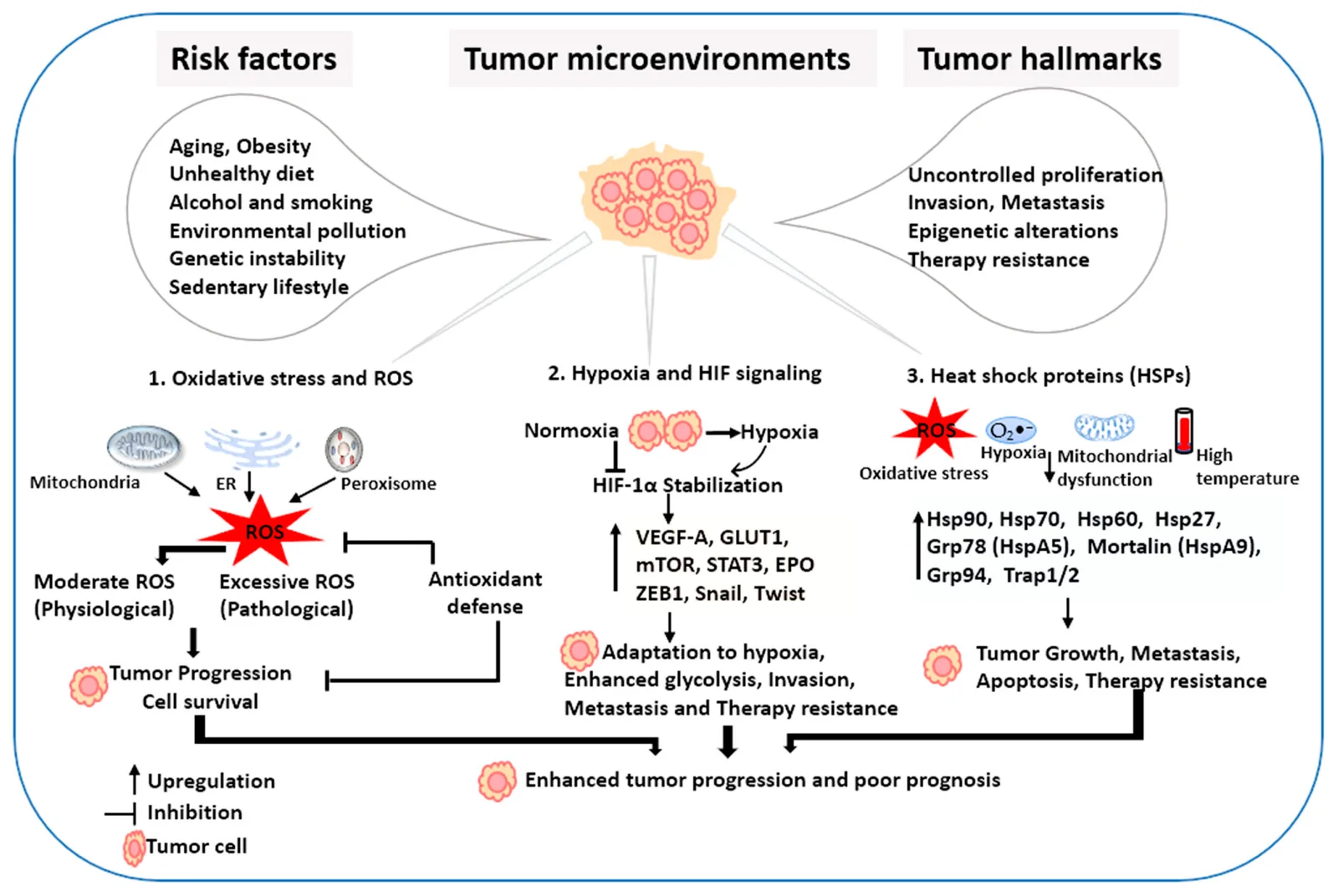
Schematic representation of the interconnected roles of oxidative stress, hypoxia, and heat shock proteins in tumor progression. Multiple cancer-associated risk factors contribute to tumor development and create a stress-adaptive tumor microenvironment. Hypoxia, ROS and HSPs act as a critical molecular crossroads, engaging in bidirectional crosstalk that promotes tumor-cell adaptation, survival, proliferation, and therapeutic resistance. Collectively, this interconnected stress-response network contributes to tumorigenesis, disease progression and poor prognosis.

In cancer cells, oxidative stress occurs due to high energy demands, which enhances oxidative phosphorylation and leads to the generation of ROS and free radicals. Within the tumor microenvironment, increased metabolic activity, particularly enhanced fatty acid oxidation and mitochondrial oxidative phosphorylation (OXPHOS), results in excessive ROS generation. Elevated levels of ROS can cause oxidative damage to proteins, nucleic acids, lipids, and cellular membranes, thereby promoting genomic instability and supporting tumor progression [3]. Simultaneously, cancer cells adapt to oxidative stress by reprogramming glucose metabolism, activating hypoxia-related signaling pathways, and enhancing the mitochondrial antioxidant system to sustain ATP production and support tumor survival. Mitochondrial (OXPHOS) and enzymatic activities in peroxisomes and the endoplasmic reticulum (ER) function as critical rheostats in various cancers through a dualistic role [4,5]. At physiological or moderate levels of ROS, including free-radical species, superoxide (O_2_•−) and hydroxyl ions (OH^−^), as well as non-radical species like hydrogen peroxide (H_2_O_2_), act as secondary messengers that activate oncogenic signaling pathways, including NRF2/KEAP1, NF-kB, PI3K/AKT, and MAPK, thereby driving cellular proliferation and tumor progression [6]. To counteract oxidative stress and maintain mitochondrial redox homeostasis, cells activate an antioxidant defense network comprising enzymes such as superoxide dismutase (SOD), catalase (CAT), and glutathione peroxidase (GPX), as well as the NRF2-mediated transcription of cytoprotective genes (Figure 1) [7]. However, when the production of ROS and reactive nitrogen species, such as peroxynitrite (ONOO^−^), overwhelms these antioxidant scaffolds, the resulting oxidative stress induces catastrophic damage to cellular macromolecules. This includes the peroxidation of polyunsaturated fatty acids (PUFAs) in cell membranes, yielding cytotoxic aldehydes such as malondialdehyde and 4-hydroxynonenal, as well as the oxidation and aggregation of proteins. Crucially, these reactive ions and free radicals cause DNA damage, characterized by strand breaks and the formation of mutagenic lesions such as 8-oxo-guanine (8-oxo-G), facilitating G→T transitions and genomic instability [8]. Mitochondrial DNA is particularly susceptible to such oxidative modifications due to the absence of protective histones and limited repair mechanisms, establishing a direct mechanistic link between mitochondrial redox imbalance and the somatic mutations driving malignancies such as breast, prostate, and colorectal cancers [9,10].

Hypoxia, a hallmark of the tumor microenvironment, is characterized by reduced oxygen availability that stabilizes hypoxia-inducible factors (HIFs), primarily HIF-1α. Activation of HIF signaling induces the transcription of a wide range of genes involved in angiogenesis, metabolism, erythropoietin (EPO), glucose transporter-1 (GLUT1), vascular endothelial growth factor-A (VEGF-A) [11], multiple glycolytic enzymes [12], and STAT3, to acclimate to hypoxic stress (Figure 1). Accumulating evidence demonstrates that hypoxia promotes tumor progression, invasion, and metastasis compared with normoxic conditions [13]. In solid tumors, oxygen delivery is frequently compromised because of rapid tumor proliferation and an expanding vascular network. Solid tumor hypoxia is typically classified into two subtypes: acute and chronic hypoxia [14,15]. Furthermore, elevated metabolic demand and increased oxygen consumption contribute to the establishment of hypoxic niches within tumors [16,17]. Under physiological conditions, oxygen homeostasis is maintained through a balance between oxygen supply and cellular demand. However, cancer cells undergo profound metabolic reprogramming to support rapid proliferation, leading to increased oxygen requirements. This metabolic adaptation, commonly referred to as the “Warburg effect,” favors aerobic glycolysis and is largely regulated by HIF-1α. In tumor cells, the induced activation of HIF-1α has been associated with ataxia-telangiectasia-mutated kinase (ATM) dependent phosphorylation and subsequent activation of the mechanistic target of rapamycin (mTOR) pathway, promoting cell survival and proliferation in several pediatric solid tumors [18]. As a master regulator of cellular adaptation to hypoxia, HIF-1α orchestrates multiple transcriptional programs that facilitate tumor growth and progression [19]. Importantly, hypoxia also contributes to metastasis dissemination by regulating epithelial–mesenchymal transition (EMT)-related transcription factors, including zinc finger E-box binding homeobox 1 (ZEB1), Snail and Twist [20]. Consequently, the hypoxic tumor microenvironment not only enhances metastasis potential but also confers resistance to chemotherapy and radiotherapy, thereby contributing to treatment failure and disease recurrence [21]. Furthermore, hypoxia also alters the expression of microRNAs (miRNAs) across cancers. HIF-1α regulates the expression of genes in signaling pathways that promote cancer cell proliferation and survival. For instance, miR-133a-3p is a hypoxia-modulated miRNA with functional consequences in prostate cancer [22]. In breast cancer MCF7 cells, miR-20b targets HIF-1α to suppress its expression [23]. Another miRNA, miR-18a, directly regulates HIF-1α expression and subsequently hypoxia-responsive genes in basal-like breast tumors [24]. These findings indicate that miRNAs are critical for hypoxia and its responsive gene expression; however, their combined effects on ROS and stress chaperones remain unclear. Therefore, further study is warranted to explore a common mechanism that leads to an integrated regulatory axis across these pathways in cancer treatment.

Another key determinant of tumor survival and adaptation is the family of molecular chaperones known as HSPs, which play essential roles in maintaining protein homeostasis under physiological and stress conditions. Tumor-associated stresses include oxidative stress, high-temperature stress, metabolic alterations, and hypoxia [25]. Cancer cells are highly dependent on HSPs because numerous oncoproteins require chaperone-mediated folding, stabilization, trafficking, and degradation for their functional activity [26]. Consequently, HSPs are frequently overexpressed in a wide range of malignancies and have been implicated in tumorigenesis, metastasis progression, and resistance to anticancer therapies [27,28]. Additionally, organelle-specific HSP homologues specifically localize to mitochondria and the ER and exert specific functions in tumor progression and growth. For instance, mortalin (HspA9), glucose-regulated protein 78 (Grp78/HspA5), Grp94, and their mitochondrial homologue, tumor necrosis factor receptor-associated protein (TRAP1/2) (Figure 1), contribute to tumor microenvironment stress adaptation. These chaperones have emerged as critical regulators of mitochondrial function, oxidative stress responses, metabolic adaptation, and cell survival regulation in cancers [29,30]. While extensive studies have traditionally focused on either the axis of ROS and hypoxia or the axis of proteotoxic stress and HSP-mediated homeostasis, these pathways are intrinsically linked. Together, they increase the stability of various client proteins critical for tumorigenesis and therapy resistance [31]. In this review, we pivot to emphasize the critical need for systematic cancer therapies specifically designed to manipulate this entire triad axis. However, we address the major clinical challenges to counterbalance the off-target effects of monotherapy inhibitors; chemotherapeutic drugs targeting these pathways often trigger resistance to many therapies. To effectively challenge this condition, a multifaceted approach targeting multiple aspects of tumor biology is essential. Such a strategy would synergistically enhance antitumor efficacy while precisely mitigating both systemic toxicity and acquired resistance mechanisms. Furthermore, we explore the critical role of HSPs in regulating cell-death pathways like ferroptosis in cancer therapeutics and maximizing clinical benefits.

## 2. Materials and Methods

This review outlines potential strategies based on hypoxia, ROS, and critical functions of HSPs in regulating these anomalies in cancer. The review encompassed the literature and research articles from the last two and a half decades, where Medline (https://www.nlm.nih.gov/medline (v2024) (2 June 2026), https://webofscience.zendesk.com/hc/en-us/articles/20138993176721-MEDLINE (2 June 2026)), PubMed (https://pubmed.ncbi.nlm.nih.gov) USA.gov, and Google Scholar (https://scholar.google.com) online free resource were used to find the most relevant and recent publications. The most important and recent research articles from each relevant author in hypoxia, ROS, and HSPs were included. The collected research allowed the inclusion of detailed information related to cancer studies across various clinical studies, focusing on molecular mechanisms and the literature related to mechanisms affecting ROS production and mitochondrial homeostasis. This review primarily focuses on hypoxia-driven stress and alterations in mitochondrial functions and HSPs in various cancers. Alterations in these important signaling pathways affect cell death pathways such as ferroptosis in various cancers as a therapeutic innovation. A detailed discussion of each section with significant mechanisms is provided, and references are cited for all encoded statements in this review.

## 3. Overview of HSPs and Their Role in Cancer

HSPs are the most representative molecular chaperones, classified based on their molecular weight into seven families, such as Hsp110, Hsp90, Hsp70, and Hsp60, and small HSPs (12–40 kDa) (Table 1) [32]. The expression of HSPs is primarily regulated by heat shock transcriptional factor (HSFs), particularly HSF-1, which undergoes trimerization, nuclear translocation, and binding to heat shock elements (HSEs) within UTRs of target gene promoters, thereby enhancing the transcription of heat shock genes [30]. The chaperone activity of HSPs is ATP-dependent, requiring ATP binding and hydrolysis to facilitate protein folding, stabilization, and quality control. In contrast, small HSPs are ATP-independent and consist of approximately 43 kDa or less in molecular weight [33]. HSPs maintain cellular protein homeostasis and participate in the proper folding, assembly, and disassembly of protein complexes [32], inhibit improper protein aggregation resulting from thermal stress, degradation, and repair of aberrant proteins [34], assemble oligomers, and transport functional proteins. Under certain viral and bacterial infections, HSPs can modulate the immune response to alleviate the infection [35,36,37]. Indeed, tumor cells rely more on HSPs than normal cells due to their supportive role in cell proliferation and survival. In malignant cells, HSPs promote tumor survival by suppressing apoptosis, attenuating antiproliferative signaling, and facilitating the refolding and functional recovery of misfolded and denatured proteins through Hsp70/Hsp90 organizing proteins (HOP), thereby preventing their degradation. These notable roles of molecular chaperones highlight two interconnected but opposite pathways: either for survival or death. The balance between these two contrasting processes depends on the cell’s capacity to sustain the stress until the cell gets back to its normal state.

Cancer cells exhibit profound metabolic reprogramming characterized by increased reliance on aerobic glycolysis to meet the heightened bioenergetic and biosynthetic demands required for growth and survival. This altered metabolic state, together with mitochondrial dysfunction, oncogenic signaling, and hypoxic stress, contributes to the excessive generation of ROS and reactive nitrogen species (RNS). While moderate levels of ROS promote tumor growth and survival, excessive oxidative stress can trigger mitochondrial damage and activate programmed cell death pathways. To counteract these deleterious effects, cancer cells rapidly induce HSPs that maintain proteostasis under stress conditions.

Furthermore, organelle-specific chaperones particularly, in mitochondria and ER emerge as a critical player in cancer progression and metastasis. For instance, Grp78 is an ER-resident chaperone primarily involved in protein folding, assembly, and trafficking while serving as a central mediator of the unfolded protein response (UPR) [47]. Upon ER stress, Grp78 coordinates adaptive signaling pathways that restore protein homeostasis and promote cellular survival. In this review, we comprehensively highlight the intricate interplay among oxidative stress, hypoxia, and HSPs-mediated adaptive responses across diverse cancer types (Table 1). Furthermore, we discuss emerging therapeutic strategies aimed at targeting this interconnected stress-response network to improve anticancer efficacy and overcome treatment resistance.

## 4. HSP Family Members and Their Role in Malignancies

### 4.1. Hsp110/HspH1 Family Chaperones

Hsp110 chaperones, also referred to as Hsp105 proteins, showed homology with canonical Hsp70. The Hsp110 family of chaperones is characterized by holdase activity, which is considered a non-canonical Hsp70 protein. The canonical Hsp70 exhibits refolding activity and is primarily involved in the folding of proteins. Similarly, Hsp110 possesses a carboxy-terminal portion, which is responsible for a peptide-binding function regulated by ATP binding and hydrolysis of its amino-terminal halves [48]. This allows these proteins to reversibly bind to other proteins and to serve as a molecular chaperone [49]. Notably, Hsp110s are marked by extended acidic insertions located within the substrate-binding domains, i.e., SBD-α and SBD-β subunits [50]. Hsp110/Grp170 families harbor unique TEDWYLEE motifs, which distinguish them from canonical Hsp70s [51]. The exclusive role of these domains has not yet been determined, but they may contribute to facilitating co-chaperone or client protein binding. ER resident stress protein Grp170, which serves as a complex with Grp78 and Grp94, exhibits sequence similarity with Hsp70 and Hsp110. Grp170 contains an ATP-binding domain and binds ATP; its C-terminus has specific NDEJ motifs, and its mRNA is anoxia-inducible [52]. Hsp110 acts as a holdase, binding and protecting unstable proteins from aggregation, and regulates the conformational Hsp70 cycle. Hsp110 and Hsp70 form a stable heterodimer and assist in disaggregation when large aggregates form [53,54].

Hsp110 has been studied in tumor immunogenicity in CT26 colorectal tumor cells, suggesting that manipulation of Hsp110 expression in tumor cells can activate antitumor immune response in vivo [55]. For instance, in colorectal cancer patients, Hsp110 is markedly increased in tumor tissues compared to adjacent normal tissues, and its expression levels negatively correlate with overall survival. Mechanistically, Hsp110 drives tumor progression by activating the Wnt/β-catenin and STAT3 signaling pathways, which inhibit apoptosis and promote cell proliferation [56]. Another study in Burkitt’s lymphoma (BL), Hsp110 supports tumor survival by regulating the expression of c-Myc, a key oncogenic transcriptional factor [57]. Hsp110 inhibition reduces c-Myc levels and impairs DNA repair pathways by downregulating essential homologous recombination (HR) and non-homologous end joining (NHEJ) proteins, including BRCA1 and Ku70 [58]. Consequently, Hsp110 silencing in primary effusion lymphomas (PEL), a rare lymphoma associated with KSHV, leads to increased DNA damage, as indicated by elevated γH2AX foci, and sensitizes tumor cells to genotoxic therapies [59]. However, the effective therapy for PEL remains not fully explored. Inhibition of Hsp110 impaired PEL cell survival through DNA damage and inhibited lysosomal permeabilization, suggesting a potential role in therapeutic intervention strategies [60]. Hsp110 generally confers cytoprotection by functioning as a stress buffer, resulting in chaperoning and anti-apoptotic activity against anticancer drugs and hypoxia [38]. Despite the therapeutic promise of targeting this protein, the field has very limited inhibitors due to a limited understanding of the protein’s binding pockets. Consequently, identifying novel Hsp110 inhibitors with high binding affinity is essential for future mechanistic studies and computer-assisted drug design (CADD). A second-generation Hsp110 inhibitor, compound 7, suppresses the STAT3 signaling pathway by downregulating STAT3 phosphorylation and thereby reducing downstream gene expression in colorectal cancer cells [61]. Additionally, Hsp110 itself could also serve as a potential tumor antigen in various cancers and correlates with the diverse stages of disease [62]. Indeed, Hsp110 DNA vaccine inhibits both CT26 colorectal cancer and B16 melanoma in mice, accompanied by activation of Hsp100-specific CD4^+^ and CD8^+^ T-cells [63].

Grp-170 (glucose-regulated protein 170) is an abundant Hsp70-like protein that resides in the ER lumen. Grp170 assists in protein folding and the transportation of secretory or transmembrane proteins. Similar to Hsp110, Grp170 also efficiently binds to ATP and triggers tumor immunity by delivering polypeptides into the ER via the transporter associated with antigen processing (TAP) [64]. In a murine model, Hsp110 was shown to activate dendritic cells (DCs), inducing the production of pro-inflammatory cytokines, including IL-6, TNF-α, and IL-12. The secreted form of Grp170 (sGrp170), by deleting the ER-retention sequence (KNDEL), showed inhibition of tumor growth in vivo through increased tumor infiltration of CD8^+^ T-cells and release of TNF-α [65]. Additionally, manipulating cellular compartmentalization from ER to extracellular milieu by deletion of ER retention signal “KNDEL” sequence of Grp170 does not change the biochemical properties of the protein [66]. Collectively, these findings highlight that Hsp110 is a critical modulator for tumor immunity and has potential as a therapeutic target in various cancers.

### 4.2. Heat Shock Protein 90 (HspC)

Hsp90 is one of the most abundant molecular chaperones, accounting for approximately 1–2% of the total soluble protein under physiological conditions. It is ubiquitously expressed and participates in numerous cellular processes, including signal transduction, protein folding and degradation, cell growth and survival, and cell death [67,68,69,70]. It facilitates signal transduction by maintaining the conformational stability and functional activation of a wide range of client proteins, including transcription factors, protein kinases, and hormone receptors [71]. Mammalian Hsp90 exists as two isoforms: the inducible Hsp90α and constitutively expressed Hsp90β, together with an additional isoform, Hsp90N, which has been implicated in cellular transformation [72]. Hsp90 isoforms are found as dimers and consist of three domains: N-terminal domain (NTD), middle domain, and C-terminal domain (CTD). In addition to its cytosolic forms, Hsp90 possesses organelle-specific homologs. For instance, glucose-regulated protein-94 (Grp94) is localized in the ER in all eukaryotes except fungi, whereas TRAP1 or TRAP2 represents the mitochondrial homolog localized within the mitochondrial matrix and shares significant sequence homology with bacterial HtpG [73]. Notably, Hsp90α can be secreted into the extracellular space, where it promotes tumor cell migration, invasion, and metastasis in various cancers such as colon cancer [74], breast cancer [75], and prostate cancer [76].

Hsp90 has emerged as a central regulator of tumor development, progression, and therapeutic resistance. Hsp90 expression is frequently elevated in a wide range of cancers, reflecting the increased demand for protein quality control resulting from the accumulation of mutated, amplified, or overexpressed oncoproteins [77]. Cancer cells exhibit enhanced dependence on Hsp90 to stabilize numerous oncogenic client proteins, thereby supporting sustained proliferation and survival. In addition to regulating oncogenic signaling, Hsp90 also modulates tumor suppressors and immune regulatory proteins, further contributing to tumor progression [78,79]. Within the tumor microenvironment, hypoxia, nutrient deprivation, and ROS further enhance Hsp90 activity, promoting immune evasion, EMT, invasion, and metastasis dissemination [80]. For instance, elevated levels of Hsp90 have been reported in approximately 90% of primary breast cancers and strongly correlate with poor prognosis [81]. Enhanced Hsp90 levels have also been observed in multiple myeloma, leukemia, colorectal cancer, non-small cell lung cancer, and prostate cancer, suggesting an important role in extracellular signaling during tumorigenesis [82]. Notably, recent reports have also observed the secreted forms of Hsp90 in various human cancers and mammalian cell lines [83].

Extracellular Hsp90-α (eHsp90α) has recently emerged as a promising pan-cancer biomarker and therapeutic target. eHsp90α promotes tumor progression by activating extracellular substrate proteins and initiating autocrine signals through cancer cell surface receptors, including low-density lipoprotein receptor-related protein-1 (LRP1) and toll-like receptors (TLR2 and TLR4) [84,85,86]. For instance, eHsp90α co-chaperone Morgana is co-secreted with Hsp90 and stimulates cancer cell migration through TLR2, TLR4, and LRP1 signaling pathways [87]. eHsp90α also promotes EMT activation by increasing the expression of EMT-associated transcription factors such as Snail (SNAI1), Slug (SNAI2), Zeb1, and Twist1, while suppressing E-cadherin expression [74,76]. In vivo studies further demonstrated that clusterin, an approximately 80-kDa glycoprotein [88], interacts with eHsp90α and potentiates LRP1-mediated signaling, thereby enhancing EMT and breast cancer cell migration [86]. Grp94, the ER-resident Hsp90 homolog, is frequently overexpressed in lung [89], gastric [90], colon [91], esophageal [92], and breast cancers [93]. Elevated Grp94 expression promotes tumor growth through activation of the Wnt/β-catenin signaling pathway, leading to increased production of pro-inflammatory cytokines and establishment of a tumor-promoting microenvironment [94]. In multiple myeloma (MM), Grp94 plays a critical role in maintaining ER proteostasis and immunoglobulin biosynthesis, highlighting its importance in plasma cell survival [95]. These findings highlight that Grp-94 represents an attractive therapeutic target alongside cytosolic Hsp90 isoforms.

TRAP1, the mitochondrial Hsp90 homolog, is also highly expressed in multiple malignancies and contributes to metabolic reprogramming by regulating mitochondrial respiration and oxidative phosphorylation [96]. Mitochondria actively participate in tumorigenesis through altered metabolism and accumulation of oncometabolites, including succinate, fumarate, and 2-hydroxy-glutrate (D-2HG). Mutations in fumarate hydratase (FH) and succinate dehydrogenase (SDH) result in the accumulation of succinate and fumarate, respectively, leading to the inhibition of prolyl hydroxylase domains (PHDs), stabilization of hypoxia-inducible factor-1α (HIF-1α), and tumor development [97]. SDH mutations are reported in hereditary paraganglioma (PGL) and pheochromocytoma (PCC), while FH mutations are reported in leiomyomatosis and renal cell carcinoma (RCC) [98]. TRAP1 suppresses SDH activity within mitochondrial respiratory complex II, thereby reducing oxidative phosphorylation while promoting succinate accumulation and HIF-1α stabilization. TRAP1 prompts a succinate-dependent inhibition of PHD, which prevents hydroxylation of HIF-1α and subsequent proteasomal degradation. This stabilization of HIF-1α promotes tumorigenesis [99]. Therefore, the induced activity of TRAP1 contributes to neoplastic growth by succinate-mediated stabilization of HIF-1α. In neurofibromin-deficient cells, ERK-dependent phosphorylation of TRAP1 inhibits SDH activity, thus enhancing succinate levels, which inhibit PDH and lead to proteasomal degradation of HIF-1α. Thus, TRAP1, ERK1/2, and SDH interaction is crucial for tumor growth [100]. Hsp90 inhibition induces HIF-1α degradation, suppresses HIF-1α dependent VEGF expression, and thus could be a potential target to inhibit cancer progression via hypoxia signaling [101]. Additionally, SDH represents a major site of mitochondrial ROS generation; TRAP1-mediated inhibition alters intracellular ROS homeostasis and enhances cancer cell survival [102]. Collectively, these findings suggest that TRAP1 functions as a critical metabolic regulator, facilitating the transition from normal cellular metabolism to the malignant phenotype.

Studies have shown that radiotherapy (RT) induces oxidative stress and metabolic remodeling in cancer cells by increasing ROS production and disrupting mitochondrial function, ultimately influencing tumor cell survival [103,104]. For instance, inhibition of glycolytic pathways, including pyruvate dehydrogenase (PDH), has been shown to enhance sensitivity to radiotherapy in NSCLC models [105]. Similarly, combined treatment with the Hsp90 inhibitor ganetespib and RT enhances T cell mediated autoimmunity in head and neck squamous cell carcinoma (HNSCC), producing greater antitumor efficiency than either treatment alone [106]. Several Hsp90 inhibitors have advanced into clinical development in various malignancies. In a phase II clinical trial, the Hsp90 inhibitor AUY922 demonstrated clinical activity in a total of 153 patients with stage IV NSCLC, particularly in tumors harboring anaplastic lymphoma kinase (ALK) rearrangements and EGFR mutations [107]. These findings support the rationale for combining Hsp90 inhibitors with targeted therapies, chemotherapy, radiotherapy, or immunotherapy to overcome resistance mediated by multiple oncogenic signaling pathways.

Over the past three decades, numerous Hsp90 inhibitors have been evaluated in combination with conventional chemotherapy, targeted agents, and immunotherapies. The underlying rationale is to simultaneously inhibit multiple survival pathways, enhance antitumor immunity, and prevent compensatory mechanisms that limit the efficacy of monotherapy. First-generation Hsp90 inhibitors, including 17-allylamino-17-demethoxygeldanamycin (17-AAG) and 17-dimethylaminoethylamino-17-demethoxygeldanamycin (17-DMAG), demonstrated promising preclinical activity but failed to achieve sustained clinical success because of poor pharmacokinetic properties, limited solubility, systemic toxicity, and induction of compensatory heat shock responses [108,109]. Although several next-generation Hsp90 inhibitors remain under clinical investigation, challenges related to toxicity, off-target effects, and activation of alternative heat shock proteins continue to limit their therapeutic efficacy [110]. Further studies should focus on optimizing dosing strategies, identifying predictive biomarkers, and developing rational combination therapies to maximize the clinical benefit of Hsp90 inhibition in both hematological and solid malignancies [111].

#### 4.2.1. Hsp90 Inhibitors

Hsp90 inhibitors have emerged as promising agents for cancer treatment due to their ability to destabilize a wide range of oncogenic client proteins. Experimental evidence demonstrated that combination therapies aim to simultaneously block multiple cancer survival pathways, enhance immune responses, and prevent compensatory mechanisms that impede monotherapy. Recent advances in drug development have led to the discovery of several Hsp90 inhibitors that have entered clinical trials and shown encouraging therapeutic potential [112]. Hsp90 inhibitors are classified depending on their binding domains: N-terminal domain (NTD)-binding inhibitors, middle domain-binding inhibitors, and C-terminal domain (CTD)-binding inhibitors. NTD-binding inhibitors are the most commonly used domain for inhibiting Hsp90 activities by blocking ATPase binding sites, thus suppressing the binding of various client proteins, and leading to their degradation. For example, geldanamycin (GA) and its derivatives, like 17-AAG, are commonly known for their anti-tumor activity. In addition to geldanamycin-type inhibitors, purine analogues such as PU-H71 also bind to NTD and significantly inhibit Hsp90 chaperone activity by suppressing ATP binding [113]. Inhibition of the NTD-ATP-binding site paradoxically triggers a compensatory heat shock response, leading to the upregulation of HSPs. This activation reduces the efficacy of drugs and requires dose adjustments that often result in significant ocular and cytotoxic side effects. Due to these limitations, many pan-Hsp90 inhibitors have failed in clinical trials, driving the development of alternative strategies. Thus, the CTD has emerged as a promising alternative target, as modulating Hsp90 activity through disrupting dimerization and allosteric modulation of the N-terminal ATP-binding site [114].

Achieving selective inhibition of specific Hsp90 isoforms remains a significant challenge in cancer therapy. Hsp90 isoform-specific inhibitors such as pimitespib, Compound 31, and SNX0723 target both cytosolic Hsp90α/β isoforms, but show weaker activities against Grp94 and Trap1 [115,116]. However, their limited ability to discriminate between the two forms remains problematic. Additionally, Hsp90α inhibition has been linked to on-target toxicities, including ocular and cardiac adverse effects. Selective inhibition of Hsp90β has emerged as a promising strategy, particularly in cancer immunotherapy, as it can modulate tumor-associated immune responses. NDNB1182, a novel Hsp90β-selective inhibitor, is being investigated for its ability to enhance innate immune signaling while minimizing the heat shock response [117,118]. These findings suggest that isoform-selective Hsp90 inhibition may offer an alternative to conventional pan-Hsp90 inhibition, although further investigation is needed to confirm its selectivity, toxicity, and clinical efficacy.

#### 4.2.2. Curcumin-Derived Hsp90 Inhibitors

Curcumin derivatives have been extensively studied for their medicinal properties. It inhibits Hsp90 activity in various cancers. For instance, the 4-arylmethyl curcumin derivative (C212) effectively eradicates both proliferating and quiescent leukemia cells, disrupting Hsp90 function [119]. Other derivatives, such as 4-(4-pyridinylmethylene) curcumin (C1206), bind to the middle domain of Hsp90 and inhibit ATPase, resulting in the degradation of Hsp90 client proteins in chronic myeloid leukemia (CML) cells [120]. However, C0818 binds to the C-terminal dimerization domain of Hsp90 and inhibits its ATPase activity [120]. Another analogue, (E)-2-(4-hydroxy-3-methoxybenzylidene)-5-((E)-3-(4-hydroxy-3-methoxyphenyl) acryloyl) cyclopentanone (CUR3d), suppresses both Hsp90AA1 and Hsp90AB1 activity, resulting in significant growth inhibition in aggressive tumors [121]. Beyond monotherapy, curcumin acts synergistically with epigenetic modulators. Notably, in vitro evidence demonstrated that the combination of curcumin with pan-HDAC inhibitors such as vorinostat and panobinostat enhances the inhibition of Hsp90 activity more effectively, leading to the profound down-regulation of several client proteins in squamous cell carcinoma and mesothelioma [122].

#### 4.2.3. Geldanamycin and Derivatives

Geldanamycin, a naturally occurring benzoquinone ansamycin, is a potent natural inhibitor of Hsp90 that competitively binds the N-terminal ATP-binding domain. Although it effectively arrests cell proliferation by disrupting chaperone activity, its clinical utility is hindered by significant hepatotoxicity. This led to the development of derivatives with improved pharmacological profiles, most notably 17-AAG and 17-DMAG, which bind to the N-terminal region and are extensively used in in vitro and in vivo experiments [123]. 17-AAG retains high anticancer activity with reduced hepatotoxicity and has demonstrated efficacy in phase I clinical trials in fifty-four eligible patients for breast cancer, lung cancer, and melanoma with significant reduction in tumor size [124,125]. However, dose-limiting toxicities such as fatigue, anemia, and elevated transaminase levels have shifted the clinical focus towards combination strategies [126]. Integrating 17-AAG with conventional chemotherapy has been shown to enhance therapeutic remission rates while maintaining manageable adverse effect profiles in both hematological malignancies and solid tumors [127,128].

17-DMAG, a water-soluble analogue of 17-AAG, offers superior bioavailability and a similar mechanism of action, including chaperone function and proteasomal clearance of Hsp90 client protein. In acute myeloid leukemia (AML) and metastatic lung cancers, 17-DMAG modulates the tumor microenvironment by suppressing the expression of HIF-1α and VEGF levels, thereby inhibiting angiogenesis and tumor progression [129]. In hepatocellular carcinoma, it triggers apoptosis via degradation of survivin, upregulation of p53, and suppression of NF-κB and cyclin D1 signaling [130]. Notably, resistance to 17-DMAG can emerge in anaplastic lymphoma kinase (ALK) rearrangement in lung cancer through increased levels of P-glycoprotein (P-gp) efflux pump, highlighting a potential challenge for sustained therapy [131].

#### 4.2.4. Purine-Based Hsp90 Inhibitors

Purine-based scaffolds represent a structurally distinct class of Hsp90 inhibitors with high affinity and favorable drug-like properties. Zelavespib (PU-H71) and CUDC-305 have demonstrated broad-spectrum activity across diverse malignancies, including lymphoma, metastatic solid tumors, and myeloproliferative neoplasms (MPNs). PU-H71 has recently shown promise in targeting AML cases characterized by the PML–SYK fusion [132]. CUDC-305 is effective across diverse models such as NSCLC, glioblastoma, colorectal cancer, and TNBC. In NSCLC models, it induces the degradation of mutant EGFR and disrupts the RAF/MEK/ERK and PI3K/AKT signaling cascades [133]. Additionally, the second-generation oral inhibitors BIIB021 and BIIB028 were investigated in early-phase clinical trials for advanced solid tumors and other malignancies; however, their clinical development was subsequently discontinued, and neither compound is currently undergoing active clinical evaluation [134].

#### 4.2.5. Next-Generation and Domain-Targeted Hsp90 Inhibitor

C-terminal-specific inhibitors, such as SL-145, epigallocatechin-3-gallate (EGCG), and HTS, disrupt the binding of key clients like JAK2/STST3, telomerase, and aryl hydrocarbon receptor (AhR) without triggering the compensatory upregulation of Hsp70 [135]. Novobiocin and its derivatives, such as KU32 and KU-596, also known as RTA 901 (developed by Reata Pharmaceuticals), are potent inhibitors of the Hsp90 C-terminus. RTA901 is the first CTD inhibitor to reach clinical trials for treatment of diabetic neuropathy. RTA 901 completed phase I and entered phase 2 trials for diabetic neuropathy (ClinicalTrials.gov ID NCT05895552) [136]. However, the clinical progression of CTD inhibitors for cancer treatment has been limited. In parallel, subcellular-specific targeting is another emerging frontier; Gamitrinib and its derivative G-G4 selectively accumulate in the mitochondria to disrupt Hsp90 and cyclophilin D complexes, showing efficacy in the prostate cancer model [137,138]. Other innovative scaffolds include Sansalvamide A-amide (San A-amide), which binds to Hsp90’s NTD-MD and allosterically disrupts HOP/Hsp90 interaction rather than inhibiting N-terminal ATPase activity. San A-amide analogs have been shown to have anticancer activity in preclinical models of pancreatic, colon, breast, and prostate cancers; however, these compounds remain at the preclinical stage and have not progressed to clinical trials [139,140]. Ganetespib, a triazolone derivative, has emerged as one of the most potent inhibitors in this case, exhibiting superior activity in drug-resistant tumors with lower hepatotoxicity and cardiotoxicity compared to ansamycins [141]. Finally, ATP-competitive inhibitors such as SNX-2112, 2-aminothieno [2,3-d] pyrimidine, and NVP-BEP800 continue to demonstrate strong oral bioavailability and potent antitumor efficacy in preclinical studies, representing a robust pipeline for future Hsp90-targeted therapies [142,143].

### 4.3. Heat Shock Protein 70 (HspA/B)

The Hsp70 family comprises highly conserved molecular chaperones that serve as the central hub of the cellular proteostasis network. These chaperones orchestrate a diverse array of functions, including the folding of nascent polypeptides, the refolding of misfolded or aggregated proteins, protein translocation across membranes, the assembly/disassembly of macromolecular complexes, and directing non-functional proteins toward degradative pathways [144]. In humans, this family is categorized into two primary functional classes: the constitutively expressed Hsc70 (Hsp73/HspA8) and the stress-inducible Hsp72 (HspA1A). Beyond the cytosol, Hsp70 isoforms exhibit strict compartmentalization: Grp78 (BIP/HspA5) resides in the ER to manage the UPR, while mortalin (Grp75/HspA9) maintains mitochondrial integrity [144]. Under environmental stress such as hypoxia, ischemia, oxidative stress, and pro-inflammatory signaling such as tumor necrosis factor alpha (TNF-α) mediated stress signaling, drastically upregulate Hsp70 levels. Notably, while intracellular Hsp72 serves as a critical cytoprotective agent against environmental proteotoxicity, its extracellular counterpart (eHsp70) functions as a potent “alarmin” or signaling molecule bridging the gap between cellular stress and the innate immune response.

In humans, the Hsp70 family comprises multiple isoforms, including HspA1A, HspA1B, HspA1L, HspA2, HspA5, HspA8 and HspA9. These isoforms exert diverse and critical functions in physiological and pathological processes, including cancer, neurodegeneration, and viral infections [145]. For instance, HspA9 promotes p53 degradation, while HspA8 stabilizes inactive p53 mutants to favor cell survival and proliferation in cancer cells [146]. These Hsp70 isoforms exert robust anti-apoptotic effects by inhibiting JNK-mediated phosphorylation and interfering with the recruitment of apoptosis protease activating factor 1 (APAF-1) and apoptosis-inducing factor (AIF), thereby stabilizing mitochondrial integrity [147,148]. Notably, HspA8 inhibits apoptosis under both overexpression and knockdown of TNF-α conditions, through its interaction with the carboxyl terminus of the Hsc70-interacting protein (CHIP) and apoptosis-regulating kinase 1 (ASK1) [149]. Beyond its intracellular roles, extracellular Hsp70 modulates innate immunity in tumor microenvironments [150]. Additionally, Hsp70 inhibitors such as Minnelide have been tested in both in vitro and in vivo models of pancreatic cancer cells. In a clinical trial of forty-five patients enrolled (23 with pancreatic cancer, 10 with colorectal cancer, and the remaining 9 with other gastrointestinal (GI) cancers), it showed anticancer activity and was recommended for a phase II evaluation [151]. Therefore, Hsp70 is targeted in various cancers, and many inhibitors, for example, 2-phenylethynesulfonamide (PES) and pifithrin-μ (and their derivatives PET-16 and PES-CI), JG-98, and VER-155008, have demonstrated anticancer activity mainly in preclinical models and have not progressed to comparable clinical development [152,153]. Moreover, isoform- and tumor-selective targeting of Hsp70 while minimizing effects on physiologically important Hsp70 family members remains a major challenge for therapeutic development.

#### 4.3.1. Hsp70 Emerging Role in Disease Therapeutics

In humans, multiple Hsp70 isoforms demonstrated stress-dependent differential expression profiles. Despite their high sequence homology, their isoform-specific roles in disease progression remain an area of intense investigation. Emerging evidence highlights the role of Hsp70 in the formation of “epichaperomes,” high-molecular-weight, specialized heterocomplexes that function as scaffolding platforms to stabilize aberrant protein–protein interactions (PPI) [154]. These epichaperomes sustain pathological states in both malignancy and neurodegeneration by rewiring cellular signaling. The assembly and activity of these complexes are further refined by post-translational modifications (PTMs), which create permissive microenvironments for disease progression [155]. While the role of Hsp90 in these complexes is well-defined, Hsp70 and its J-domain co-chaperones provide the necessary spatial and temporal specificity for epichaperome functions. Consequently, targeting Hsp70 has become a major therapeutic objective; however, the high sequence homology between isoforms presents a significant challenge for developing selective small-molecule inhibitors.

#### 4.3.2. Mechanistic Contributions of Hsp70 to Tumorigenesis and Metastasis

In many malignancies, Hsp70 serves as a critical driver of “non-oncogenic addiction.” Its upregulation establishes a cytoprotective buffer that suppresses apoptosis, facilitates cell proliferation, and confers resistance to conventional chemotherapeutics [156]. Clinically, elevated Hsp70 expression correlated with aggressive disease characteristics, including lymph node metastasis and diminished overall survival in breast and cervical cancer [157,158]. In the tumor microenvironment, Hsp70 is frequently localized to the plasma membrane or secreted via exosomes and extracellular vesicles (EVs). This eHsp70 modulates immune surveillance in a context-dependent manner. While acute exposure to eHsp70 can prime anti-tumor immunity, chronic elevation of Hsp70 typically leads to immune desensitization and tumor evasion [159]. For instance, elevated serum Hsp70 levels have emerged as a robust diagnostic and prognostic “liquid biopsy” biomarker, validated across non-small cell lung cancer (NSCLC) [160], acute lymphoblastic leukemia (ALL) [161], colorectal cancer [162], gastric cancer [163], pancreatic cancer [164], and breast cancer [165]. In pancreatic cancer and NSCLC, Hsp70 negatively regulates AMPK signaling, thereby modulating autophagy flux to meet the metabolic demands of the tumor. Dual inhibition of Hsp70 and autophagy pathways yields synergistic anti-tumor efficiency [166,167,168]. Hsp70 levels correlate strongly with metastasis potential, particularly in breast cancer and MCF-7 cells [157]. Hsp70 accelerates G0/G1-to S-phase transitions, conferring both proliferative advantages and resistance to hyperthermia [169]. The mitochondrial isoform, mortalin (HspA9), is frequently overexpressed in gastric, hepatic, and breast cancers [170,171,172]. Mortalin physically sequesters the tumor suppressor p53 in the cytoplasm, thus abrogating its nuclear transcriptional activity and pro-apoptotic functions [173,174].

Similarly, the HspA8 isoform maintains proteostasis by facilitating chaperone-mediated autophagy (CMA) and stabilizing the Cyclin D1/CDK4 complex to drive cell cycle progression [175,176]. HspA8 also interacts with chromatin assembly factor 1 (CAF1) to modulate histone H3.1 dynamics, linking lipid signaling to epigenetic regulation [177]. In endometrial carcinoma, HspA8 knockdown suppresses growth via S-phase arrest, highlighting its potential as a therapeutic vulnerability [178]. Conversely, inducible Hsp70 can elicit potent anti-tumor immune responses under high-temperature stress, priming CD8+ T cells and activating immune responses through antigen-presenting cells [179]. Considering its dual role in various cancer types, its elevated level in serum may serve as a viable biomarker for diagnosis and a potential target for cancer therapies [180].

### 4.4. Hsp60: A Pleiotropic Chaperonin in Proteostasis and Diseases

Hsp60 is a nuclear-encoded mitochondrial chaperonin essential for cellular proteostasis. It is predominantly localized in mitochondria (80–85%); a significant fraction (15–20%) resides in the cytoplasm, nucleus, and on the cell surface [43,181]. Beyond its canonical “foldase” activity, Hsp60 modulates stress signaling, mitochondrial integrity, immune response, and pro- and anti-apoptotic functions. In the cytosol, it interacts with IKK complexes to modulate NF-kB-mediated antioxidant responses, protecting cells from oxidative stress-induced death [182,183,184]. Hsp60 dysregulation is a hallmark of diverse pathologies, ranging from type-2 diabetes, hepatitis B, cardiovascular disease, atherosclerosis, periodontitis, juvenile idiopathic arthritis to various cancers [185,186,187,188,189]. Furthermore, Hsp60’s role has been demonstrated in cell signaling, endothelial stress activation, and cardiovascular diseases, where it often exerts context-specific, dualistic effects; thus, it is considered a potential therapeutic target in diverse tumor and cancer cells [188,190]. Mechanistically, Hsp60 promotes neuroinflammation in diabetes by activating microglia and astrocytes through the release of inflammatory cytokines IL-1β, IL-6, and TNF-α via ERK-1/2, JNK, and NF-κB signaling [191]. Conversely, it exhibits neuroprotective properties by sequestering Aβ oligomers, thereby mitigating mitochondrial dysfunctions [192,193,194,195]. Together, these multifaceted roles underscore Hsp60’s significance in disease pathogenesis and therapeutic intervention.

#### Hsp60 and Emerging Role in Cancers

Hsp60 is markedly overexpressed in a wide array of malignancies, including colorectal cancer (CRC), NSCLC, breast cancer, hepatocellular carcinoma (HCC), oral cancer, and esophageal squamous cell carcinoma (ESCC) [196]. Conversely, its downregulation in bladder and clear cell renal carcinomas suggests that Hsp60 function is recalibrated by specific metabolic microenvironments, where it may paradoxically exert tumor-suppressive effects [197]. In contrast, Hsp60 remains non-dysregulated in skin cutaneous melanoma (SKCM), thyroid carcinoma, and thymoma. In neuroblastoma, the expression of Hsp60 is upregulated and stabilizes mitochondria survivin, a potent inhibitor of apoptosis, that subsequently facilitates tumor cell longevity [198]. Hsp60 sequesters pro-apoptotic Bax and Bak in the cytosol, preventing their translocation to the mitochondrial outer membrane and the subsequent release of cytochrome C [199]. Conversely, loss of Hsp60 proteostasis has been shown to stabilize p53, triggering p53-dependent apoptotic cascades, which underscores the protein’s role in buffering cells against tumor-suppressor activity [200]. Beyond its intracellular roles, Hsp60 is actively trafficked via EVs and exosomes into the systemic circulation. In papillary thyroid carcinoma (PTC), colon cancer, and benign goiter (BG), circulating exosomal Hsp60 levels correlate precisely with tumor burden and decline significantly following surgical resection [192]. These findings position Hsp60 not only as a mediator of peritumoral communication but also as a high-fidelity ‘liquid biopsy’ biomarker for longitudinal disease monitoring.

In CRC, the depletion disrupts mitochondrial proteostasis, leading to an aberrant accumulation of adenine. This metabolic shift triggers the AMPK signaling pathway, which acts as an energy sensor to suppress protein translation and halt cell proliferation [201]. In prostate cancer, Hsp60 maintains an aggressive phenotype by interacting with the mitochondrial protease ClpP. Recently, it has been demonstrated that small-molecule inhibitors like DCEM1 disrupt these Hsp60-ClpP interactions, resulting in impaired mitochondrial function, suppressed Myc signaling, and diminished ATP production, effectively limiting tumor recurrence and metastasis potential [202]. In HCC and oral squamous cell carcinoma, mitochondrial Hsp60 physically stabilizes the survivin complex. Pharmacological intervention with mifepristone, a progesterone and glucocorticoid receptor (GR) antagonist, has been shown to induce G1-phase arrest and apoptosis by promoting the ubiquitin-proteasomal degradation of survivin and forcing the translocation of Hsp60 to the cytoplasm and trigger apoptosis [203,204].

Hsp60 extends its influence into the tumor microenvironment by engaging TLRs and modulating the STAT3 and NF-kB signaling axes. In nasopharyngeal carcinoma, disrupting these survival signals through a combination of the PI3K inhibitor PQR309 and gemcitabine demonstrates synergistic anti-tumor efficacy [205]. However, in ovarian cancer, the role of Hsp60 remains unexplored. Some in vitro studies suggest that Hsp60 knockdown in ovarian cells disrupts mitochondrial homeostasis and paradoxically enhances cell proliferation and invasion [206]. Proteomic analysis suggests that alterations in Hsp60 phosphorylation states modulate acyl-CoA dehydrogenase (ACAD) activity, leading to dysfunctional fatty acid oxidation (FAO) and shifted energy dynamics [207,208]. Furthermore, Hsp60 contributes to an immunosuppressive milieu by regulating pro-inflammatory cytokine production. Inhibitors such as mizoribine and the IRE1α disruptor KIRA6 target the Hsp60-IKKβ axis, thereby suppressing the release of pro-inflammatory cytokines IL-1β, IL-6, and TNF-α. This inhibition also stimulates CXCL8-mediated immune evasion, suggesting that Hsp60-targeted therapies could potentially “warm up” cold tumors and enhance the efficacy of immunotherapy.

### 4.5. Small Heat Shock Proteins (sHSPs)

sHSPs represent an evolutionarily conserved yet highly divergent family of molecular chaperones ubiquitous across all organisms. Unlike their larger ATP-dependent counterparts, sHSPs exhibit remarkable sequence heterogeneity at both the nucleotide and amino acid levels. While intra-specific conservation is relatively high, inter-specific diversity is a hallmark of the family [209,210]. In humans, the sHSPs family comprises eleven members, defined by a signature conserved α-crystalline domain (ACD), which is flanked by intrinsically disordered, variable N- and C-terminal motifs. Among these, Hsp27 (HspB1), αA-crystalline (HspB4), and αB-crystalline (HspB5) are the most extensively characterized. Structurally, the ACD consists of approximately 80 to 100 amino acid residues organized into a classical β-sandwich fold of seven or eight antiparallel β-strands [211]. These low-molecular-weight monomers typically assemble into dynamic, high-order oligomeric complexes, ranging from 12 to 42 subunits [210]. This oligomerization, critically mediated by the C-terminal domain, is essential for the chaperone activity required to maintain cellular proteostasis [212,213,214]. Such conformational plasticity allows sHSPs to recognize and sequester a diverse repertoire of misfolded client proteins, thereby preventing irreversible aggregation under conditions of acute proteotoxic stress.

Hsp27 (HspB1) stands out as a potent regulator of programmed cell death, modulating both the intrinsic and extrinsic apoptosis pathways. Its cytoprotective capacity is dictated by a sophisticated interplay between its phosphorylation status and its quaternary structure. In its unphosphorylated state, Hsp27 exists as large oligomers that sequester cytochrome c, thereby thwarting the assembly of the pro-apoptotic apoptosome. Upon phosphorylation, Hsp27 dissociates into smaller dimers that interact with DAXX, inhibiting Fas-mediated extrinsic apoptosis [215,216]. Beyond these nodes, Hsp27 suppresses the activation of the pro-apoptotic BCL-2 family member Bid, shielding the cell from caspase-8-dependent death signals. Furthermore, Hsp27 functions as a molecular scaffold for pro-survival serine/threonine kinases and the actin cytoskeleton. By stabilizing microfilament integrity, Hsp27 is a pathological driver across a broad spectrum of human diseases, ranging from myopathies and cataracts to neurodegenerative disorders such as Alzheimer’s disease and multiple sclerosis [217,218,219].

The physiological versatility of the sHSPs family is further exemplified by members like HspB2, which localizes to the outer mitochondrial membrane in cardiac and skeletal muscles [220]. However, in the context of oncology, Hsp27 emerges as a critical mediator of tumor progression, metastasis, and the evasion of apoptosis. Elevated Hsp27 expression is a prognostic hallmark of several malignancies, including breast and endometrial cancers, as well as specific leukemic lineages [221,222]. In breast cancer, Hsp27 overexpression facilitates anchorage-independent growth, enhances invasive potential, and confers multidrug resistance [223,224,225]. The clinical significance of Hsp27 is perhaps most pronounced in prostate cancer (PC), where its levels surge following androgen ablation therapy. In PC, Hsp27 serves as a key molecular switch facilitating the transition to lethal castration-resistant prostate cancer (CRPC) [226,227]. The multifaceted cytoprotective utility of Hsp27 in malignant cells involves direct inhibition of caspase activation, the modulation of intracellular oxidative stress, and the stabilization of the cytoskeleton to support cell motility and EMT [228,229]. Given its central role in orchestrating survival signaling and therapeutic recalcitrance, Hsp27 has transitioned from a mere stress marker to a high-priority therapeutic target and a vital biomarker for advanced cancer diagnosis.

Hsp27 is an ATP-independent molecular chaperone that promotes tumorigenesis and therapeutic resistance by suppressing apoptosis and stabilizing the cytoskeleton [230]. Pharmacological targeting of Hsp27 is challenging due to the lack of a canonical ATP-binding pocket. However, many Hsp27 inhibitors have been evaluated that disrupt its dimerization and oligomerization [231]. Current therapeutic strategies primarily focus on three main classes of inhibitors: small molecules, peptide aptamers, and antisense oligonucleotide (OGX-427). Small molecules such as bromo-vinyl-deoxyuridine (BVDU), J2, and quercetin demonstrated significant activity in clinical studies but were discontinued due to overlapping toxic effects associated with gemcitabine in patients [232,233]. Peptide aptamers, including PA11 and PA50, represent another approach and interfere with Hsp27 quaternary structure to negatively regulate its function [232]. However, a second-generation antisense oligonucleotide, apatorsen (OGX-427), to degrade Hsp27 mRNA, has undergone extensive clinical evaluation. A phase I study in 42 patients with pancreatic cancer showed positive results with decreased Hsp27 levels [234]. Another study in 99 patients showed that OGX-427 combined with docetaxel had significant effects in urothelial cancer, increasing survivability [235]. Conversely, it failed to improve survival in a phase II trial involving non-squamous cell carcinoma or metastatic castration-resistant prostate cancer [236]. Another in silico study identified that the compounds XAV-939 and FR180204 have significant anti-Hsp27 activity and need to be evaluated in vitro and in vivo, as well as in early-stage clinical trials; thus, they could be a promising strategy in chemotherapy-resistant cancers [231,237].

## 5. Hypoxia, ROS, and HSPs Interaction Networks in Cancers

**Figure 2:**
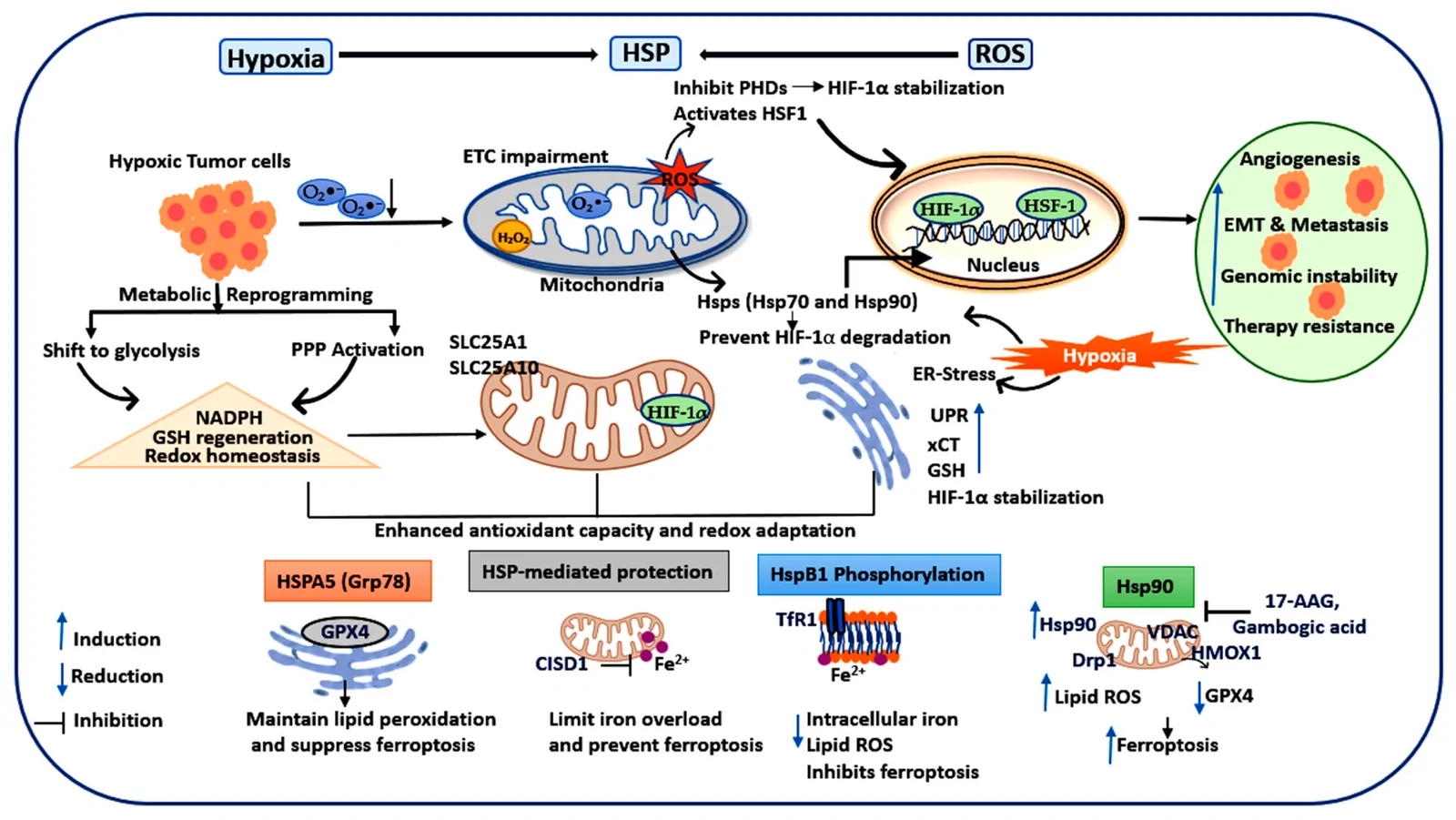
Hypoxia, Mitochondrial ROS, and HSP crosstalk in cancer. The schematic image represents that HSPs coordinate between the hypoxic-microenvironment-induced metabolic reprogramming and ROS regulation via Hsp70 and Hsp90. Furthermore, hypoxia-induced ER stress and mitochondrial ROS contribute to HIF-1α stabilization and cellular adaptation. HSPs also regulate ferroptosis through multiple mechanisms, including lipid peroxidation and iron homeostasis, by maintaining GPX4 activity and the antioxidant system. Pharmacological inhibition of Hsp90 disrupts these protective mechanisms, promoting ferroptosis-mediated cell death.

Hypoxia is a hallmark of the solid tumor microenvironment, where limited oxygen supply and high metabolic demand promote increased mitochondrial ROS and metabolic reprogramming, which drive enhanced tumor aggressiveness, metastasis, and resistance to conventional therapy, including chemotherapy, radiotherapy, and immunotherapy. In aggressive cancers, enhanced metabolic stress and suppressed antioxidant capacity trigger hypoxia-induced mitochondrial ROS, which act as critical signaling rheostats, stabilizing HIF-1α by inhibiting PHDs [238]. This metabolic shift is further fortified by increased expression of HSPs, specifically Hsp70 and Hsp90, which shield HIF-1α from proteasomal degradation, thereby sustaining pro-tumorigenic transcriptional activity [239]. HIF-1α stabilization activates transcription of target genes, such as VEGF, that facilitate the angiogenic process, hence supporting cancer cells’ resilience to treatment [240]. Recent evidence characterizes this interplay as a bidirectional feed-forward loop: hypoxia-induced mitochondrial ROS trigger the activation of HSF1 via mitochondrial perinuclear clustering, allowing the heat shock response (HSR) components and HIF-1α to synergistically regulate antioxidant defenses and the epithelial–mesenchymal transition (EMT) (Figure 2) [241]. Conversely, impaired electron transport chain (ETC) flux during hypoxia exacerbates ROS generation, which promotes genomic instability and primes the cell for oxidative damage upon reoxygenation [242].

Hypoxia orchestrates a metabolic switch from OXPHOS to glycolysis and the pentose phosphate pathway (PPP), the latter of which is essential for maintaining redox homeostasis [243,244]. For instance, HIF-1α-mediated induction of pyruvate kinase M2 (PKM2) enhances transcription of metabolic genes through its translocation into the nucleus under hypoxic conditions via Jumonji C domain-containing dioxygenase (JMJD5) [245]. Thereby, JMJD5 function is recognized as an oncogene, in part by regulating cellular metabolism through PKM2 and HIF-1α. PKM2 expression is upregulated in several cancers, including lung, breast, blood, prostate, cervix, kidney, bladder, and colon, compared to normal tissues [246]. This upregulated PKM2 is hydroxylated at proline-403/408, promoting its interaction with PHD3 and subsequent binding to HIF-1α. This positive feedback loop promotes sustained HIF-1α transactivation and reprograms glucose metabolism in cancer cells [247]. In aggressive malignancies like glioblastoma, lung, and prostate cancers, severe cyclic hypoxia upregulates mitochondrial metabolite carriers, SLC25A1 and SLC25A10, further augmenting NADPH levels and fatty acid metabolism to confer radiotherapy resistance (Figure 2) [248,249]. Beyond its canonical transcription role, HIF-1α can translocate directly to the mitochondria to attenuate oxidative stress and protect against irradiation-induced apoptosis in renal carcinoma cells [250]. Furthermore, severe hypoxia-induced ER stress activates the UPR through the PERK/eIF2α/NRF2 signaling axis, which upregulates the glutamate-cystine antiporter (xCT) expression, promoting GSH synthesis and stabilizing HIF-1α in pancreatic and lung cancers, promoting resistance to radiotherapy [251,252]. Together, these integrated pathways establish hypoxia as a core factor modulating ROS, oxidative stress, and metabolic resilience of aggressive malignancies.

To survive the hypoxia-induced ROS surge, cancer cells leverage the chaperone machinery to evade ferroptosis- an iron-dependent form of regulated cell death driven by ROS and lipid peroxidation. Hypoxia-induced mitochondrial OXPHOS, which results in mitochondrial antioxidant enzyme activation, exacerbates genomic instability, tumor aggressiveness, and resistance to chemotherapy. ROS activates the oxidation of PUFAs in the cell membrane, triggering lipid peroxidation. Without an adaptive response, the hypoxic cell would undergo rapid ferroptotic collapse. Under hypoxic stress, the ER-resident chaperone HspA5 (Grp78) is induced to stabilize GPX4, the primary quencher of lipid peroxides, thereby preventing its ubiquitin-mediated degradation, which suppresses ferroptosis and cancer cell death [253]. In pancreatic ductal adenocarcinoma (PDAC), HspA5 negatively regulates ferroptosis through activating transcription factor 4 (ATF4), maintaining GPX4 levels to sustain tumor viability (Figure 2) [254]. Similarly, dihydroartemisinin (DHA) induces ferroptosis in glioma cells by ROS generation and lipid peroxidation [255]. DHA treatment induces the expression of HspA5 via the PERK-ATF4 pathway and enhances the expression of GPX4, which subsequently attenuates the sensitivity of glioma cells to ferroptosis. These findings suggest DHA plays an anticancer role through a negative feedback loop mediated by ER stress.

Furthermore, HspB1 is phosphorylated in response to ROS, protecting cells by downregulating transferrin receptor 1 (TfR1)-mediated cellular iron uptake that reduces the intracellular level of iron through stabilization of the cortical actin cytoskeleton rather than the classical IRP/IRE pathway [256]. While HspB1 overexpression confers resistance to ferroptosis-inducing agents such as erastin, its inhibition sensitizes tumors in human xenograft mouse tumor models [257]. These findings demonstrate HspB1 as a negative regulator of ferroptosis by reducing cellular iron uptake and lipid ROS production, thus considering it as a potent therapeutic target in cancers, where erastin induces lipid ROS [258]. A mitochondrial protein, CDGSH iron sulfur domain 1 (CISD1), localized in the outer membrane, negatively regulates ferroptosis by limiting mitochondrial iron accumulation; however, its potential crosstalk with the HSP network remains a compelling yet unexplored frontier in hepatocellular carcinoma and breast cancer research (Figure 2) [259].

Hsp90 is a master regulator of oncogenic protein folding, and its role in ferroptosis is increasingly recognized as context-dependent. Although Hsp90-targeted monotherapy has shown promising antitumor activity, its clinical efficacy is frequently constrained by adaptive stress responses and limited therapeutic windows, supporting the development of rational combination strategies. In this context, ROS represent a critical mechanistic link between Hsp90 signaling, redox homeostasis, and regulated cell death. While moderate ROS levels can promote tumor adaptation and survival, excessive ROS accumulation disrupts redox equilibrium and can trigger apoptosis, senescence, and cell death, specifically, ferroptosis [260]. Hsp90 exhibits a dual role in ferroptosis. Hsp90 overexpression, together with calcineurin (CN)-dependent activation of Drp1, has been shown to promote erastin-induced ferroptosis by altering mitochondrial morphology and facilitating the accumulation of lipid ROS [261]. Conversely, Gambogic acid, an Hsp90 inhibitor, reduces GPX4 levels, thus disrupting intracellular redox homeostasis and promoting ferroptosis [262]. In NSCLC, inhibition of the Hsp90-NTD with 17-AAG increased GPX4 expression and attenuated Timosaponin AIII-induced ferroptosis, suggesting that NTD-directed Hsp90 inhibition can activate compensatory antioxidant mechanisms that restrain ferroptosis signaling [263]. In contrast, selective inhibition of Hsp90-CTD promotes the release of heme oxygenase-1 (HMOX1) into the cytoplasm via VDAC1 oligomerization, resulting in iron overload and ultimately promoting ferroptosis (Figure 2) [264]. Collectively, these establish Hsp90 as a context-dependent modulator of ferroptosis rather than a unidirectional regulator, with distinct Hsp90 domains and client proteins. This mechanistic complexity provides a strong rationale for combining Hsp90-directed agents with ferroptosis-induced therapy to sensitize resistant tumor cells and enhance therapeutic outcomes [265].

## 6. Major Challenges and Translational Limitations of Triad Axis

**Figure 3:**
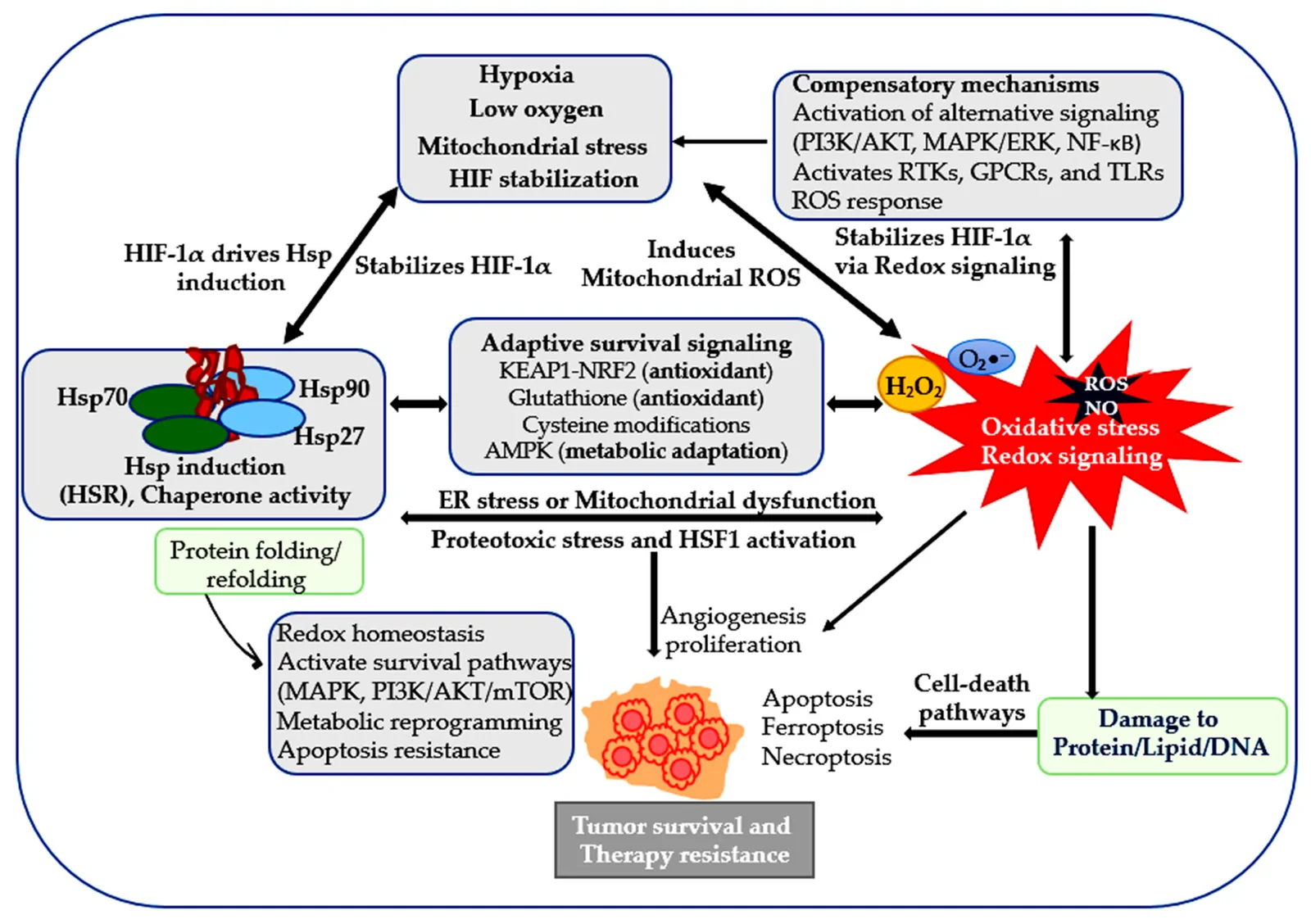
Integrated hypoxia–ROS–HSP crosstalk and compensatory mechanisms underlying tumor survival and therapy resistance. The figure illustrates the bidirectional interdependence of the triad signaling in tumor cells. Therapeutic strategies that target a single factor (e.g., hypoxia) are often compensated for by the activation of other pathways (e.g., HSR activity). This establishes a feed-forward loop in which low oxygen levels produce ROS, which then amplify the hypoxia response, leading to further metabolic reprogramming and angiogenesis. Additionally, ROS accumulation triggers oxidative damage, excess lipid peroxidation, protein modifications, and DNA damage, leading to cell-death signaling, including apoptosis, ferroptosis, and necroptosis. The activated HSR stabilizes the HIF-1α complex, allowing the tumor to survive under severe metabolic and oxidative stress. To effectively intervene therapeutically, it may be necessary to disrupt multiple points within this triad simultaneously to overcome the adaptive capacity of the tumor cells.

Despite the advancements in cancer therapy, eradicating tumors in advanced stages remains a primary clinical challenge, largely due to the development of therapeutic resistance and tumor dormancy. Therefore, combination therapy targeting the hypoxia–ROS–HSP pathways subsequently enhances efficacy in preclinical trials. The tumor microenvironment, characterized by pathological hypoxia, is a critical driver of therapy resistance. Hypoxia alters cancer cell metabolism and activates complex pro-survival pathways that contribute to therapy resistance [266]. Hypoxia induces a number of complex intracellular signaling pathways such as HIF signaling and associated cascades such as PI3K/AKT/mTOR, MAPK/ERK, and NF-κβ [267,268,269]. Notably, HIF-1α stabilization can also occur in a hypoxia-independent manner, mediated by diverse stimuli including nitric oxide (NO), ROS, cytokines, lipopolysaccharides, and growth factors through receptor tyrosine kinases (RTKs), G protein-coupled receptors (GPCRs), and toll-like receptors (TLRs) (Figure 3). Collectively, these adaptive pathways facilitate tumor cell survival by modulating hypoxia-related signaling that facilitates cell survival and propagation of tumors [266]. Conversely, PI3K/Akt signaling associated with mitochondrial ETC modulation and reduction in antioxidant defense mechanisms facilitates tumor cell survival [270].

In this context, ROS play a paradoxical role, acting as either signaling molecules that promote proliferation and adaptation or, at higher concentrations, as cytotoxic agents inducing cell death [271]. HSPs are integral to managing this redox balance, functioning in both redox buffering and stress-response signaling. However, it remains unclear how individual HSP family members differentially regulate localized ROS signaling versus global oxidative injury [272]. Mitochondrial-driven ROS specifically triggers adaptive responses, including KEAP1-NRF2 antioxidant signaling, AMPK-linked metabolic adaptation, and HIF-1α stabilization [273,274]. Conversely, severe mitochondrial dysfunction leads to overwhelming oxidative stress, resulting in lipid peroxidation, protein oxidation/carbonylation, DNA damage, and activation of regulated cell-death pathways such as apoptosis, ferroptosis, and necroptosis [275,276]. In contrast, Hsp27, as an ATP-independent chaperone with holdase activity, protects cells from oxidative stress via a glutathione-dependent antioxidant mechanism [277]. Additionally, Hsp70 also contributes to redox protection through cysteine modifications associated with NRF2/HO-1-driven antioxidant responses [278,279]. Hsp90 further supports antioxidant adaptation through stabilization of HIF-1α, which integrates hypoxic and oxidative-stress pathways, including NRF2-associated responses, to regulate mitochondrial and antioxidant homeostasis [280]. Thus, manipulating ROS levels through HSP inhibition, specifically when combined with ROS-inducing agents like doxorubicin, paclitaxel, or radiotherapy, may enhance ROS beyond the antioxidant buffering capacity of cancer cells and trigger oxidative cell death, including apoptosis and ferroptosis (Figure 3) [281]. Although rare for Hsp90 itself, mutations in co-chaperones or the emergence of resistant HSP isoforms can reduce binding affinity for inhibitors. Furthermore, activation of alternative survival pathways can bypass recruitment of the inhibited triad component [282]. Additionally, HIF inhibitors may also target hypoxia-associated signaling but escape hypoxic-independent ROS responses, which may be compensated by tumor adaptive responses [281]. Therefore, rational combination strategies designed to reshape the redox state and resolve ROS in cancer cells warrant further investigation to overcome resistance and improve outcomes in advanced cancer [283].

## 7. Conclusions and Future Direction

Collectively, the evidence discussed in this review highlights a highly interconnected stress-adaptation network in which oxidative stress, hypoxia, and HSPs are not isolated mechanisms but mutually reinforcing regulators of tumor progression and therapeutic resistance. Under stress conditions, metabolic reprogramming and mitochondrial dysfunction increase ROS production, while hypoxia-driven HIF signaling reshapes cellular metabolism, redox homeostasis, invasion, and survival. In parallel, HSPs maintain proteome homeostasis and stabilize key oncogenic factors and stress-responsive proteins, thereby enabling cancer cells to withstand the metabolic, oxidative, and hypoxic pressures imposed by the metabolic reprogramming. Notably, these pathways exhibit extensive bidirectional crosstalk; ROS can modulate HIF and HSPs response in cellular organelles, which are able to regulate cancer pathology. This integrated stress response network provides cancer cells with remarkable phenotypic plasticity, facilitating tumor initiation, metastasis dissemination, immune evasion, and resistance to conventional and targeted therapies. Therefore, disrupting individual stress pathways may be insufficient because compensatory activation of interconnected mechanisms can preserve tumor cell survival. They require a coordinated resolution once stress is removed to restore cellular homeostasis. Further therapeutic strategies should consequently focus on targeting the molecular nodes and feedback circuits linking ROS, HIF signaling, and HSPs-mediated proteostasis, potentially through combination approaches that simultaneously perturb redox balance, hypoxic adaptation, and chaperone-driven cellular adaptation to regulate cell-death pathways like ferroptosis.

HSPs exhibit a dual role in modulating cell death pathways. For instance, Hsp90 and Hsp70 attenuate apoptosis by stabilizing HIF signaling, lipid peroxidation, and iron homeostasis, thereby inducing tumor cell survival and metastasis [284]. A deeper understanding of tumor- and context-specific differences in this crosstalk, together with the identification of predictive biomarkers, will be essential for translating these mechanistic insights into effective precision therapies and overcoming treatment resistance. Given these complexities, combination therapy targeting multiple pathways such as hypoxia and oxidative-stress-induced free radicals and mitochondrial OXFOS has shown promise in improving clinical outcomes. However, the efficiency of such regimens is contingent upon patient physiological conditions, immune status, and drug metabolism. Ultimately, targeting the HIF-ROS-HSPs triad axis may provide a promising framework for developing next-generation anticancer strategies that exploit the intrinsic vulnerabilities created by the hostile tumor environment. The success of triad-targeted strategies relies on identifying patients whose tumors are screened under these pathways. Such multidimensional screening may be more reliable for combination therapy than single pathway-based selection. Currently, robust predictive biomarkers that correlate with responses to HSPs or specific hypoxia inhibitors are lacking, leading to unselected patient populations and diluted efficacy signals in preclinical trials. Further translational success will depend on developing agents that can simultaneously block redundant compensatory pathways such as HSPs, ROS, and redox homeostasis that support the tumor microenvironment. Additionally, to mitigate potential off-target toxicity and adverse physiological effects, rigorous preclinical dose optimization and robust toxicity assays remain imperative.

## Figures and Tables

**Table 1:** HSPs: cellular localization and biological functions in cancer.

HSPs	Cellular Localization	Biological Functions and Relevance to Cancer	HSPs Function in Hypoxia and ROS	References
High molecular weight chaperones (Hsp110)	Cytoplasm, nucleus, endocytic vesicles, extracellular vesicles/exosome, and ER	Prevent the aggregation of misfolded or denatured proteins and facilitate their refolding or degradation. Elicits humoral immune responses in leukemia patients. Implicated in suppression of apoptosis and aggregation, thus promotes cell survival.	Hsp110 is less studied in the context of hypoxia and ROS; thus, investigation in tumors and cancers is needed.	[38]
Hsp90	Cytoplasm and nucleus	Frequently overexpressed in various cancers, including breast cancer, pancreatic cancer, lung, endometrial, oropharyngeal squamous cell cancer, and multiple myeloma, facilitates signal transduction.	Hsp90 interacts with and stabilizes HIF-1α, thereby sustaining hypoxia-responsive transcription and VEGF-dependent angiogenesis. It also contributes to redox adaptation and can influence ROS-sensitive signaling pathways. Conversely, oxidative stress can increase chaperone activity, thus forming reciprocal HSP-ROS adaptation.	[39,40]
Hsp70	Cytoplasm and nucleus, also detected in mitochondria and extracellular vesicles under specific stress conditions	Involved in protein folding, refolding of proteins. Frequently upregulated in various cancers including oral, liver, prostate, colorectal, lung, uterine, cervical, renal cancers, and melanoma.	HIF-1α directly binds hypoxia-responsive elements in the Hsp70-2 promoter, increasing Hsp70 expression and resistance to hypoxia-induced apoptosis. Hsp70 also contributes to cellular protection against oxidative stress and therefore forms part of the adaptive response to ROS.	[41]
Hsc70	Predominantly cytoplasm and nucleus	Constitutively expressed molecular chaperone involved in protein folding, trafficking and proteostasis. Upregulated in colon and esophageal cancers.	Hsc70 contributes primarily to proteostasis during cellular stress, including oxidative and metabolic stress. Its cooperation with Hsp110 and the Hsp70-related chaperone system may help cancer cells tolerate hypoxia-associated proteotoxic stress.	[41]
Hsp60	Primarily mitochondria, also detected in cytoplasm and nucleus under pathological conditions	Aberrant expression has been implicated in tumorigenesis, invasion, and metastasis in various cancers such as breast, liver, lung, colorectal, gastric, pancreatic, adrenocortical, ovarian, prostate, bladder, cervical, head and neck cancers. Altered Hsp60 expression has also been associated with replicative senescence and stress adaptation.	Hsp60 is primarily involved in mitochondria-driven ROS and hypoxia-associated dysfunction. Hsp60 can buffer oxidative stress and protect mitochondria from ROS-mediated injury, whereas altered Hsp60 expression can contribute to metabolic reprogramming and redox adaptation.	[42,43]
Hsp40/DNAJ	Cytoplasm, ER, nucleus, and other cellular compartments depending on the family members	Primarily acts as co-chaperone of Hsp70 and regulates substrate recognition. Dysregulated expression has been reported in various cancers including breast, colon, lung, and ovarian cancers. It promotes the EMT, metastasis, and angiogenesis. Some cases it showed the inverse function such as reduced invasion, tumor migration and induced apoptosis.	Hsp40 contributes to the Hsp70-centered stress response, thereby potentially influencing cellular adaptation to hypoxia and oxidative/proteiotoxic stress. Individual DNAJ proteins can have distinct, and sometimes opposing, effects on tumor progression; therefore, their activity is primarily linked to isoform and context, rather than generalized across the family.	[44]
Small heat shock proteins (sHSPs)	Predominantly cytoplasm and nucleus	Acts as ATP-independent chaperones that stabilizes partially unfolded protein and aggregation. sHSPs has been reported in EMT, cell migration, invasion, and metastasis. Upregulated in AML, astrocytoma, thyroid, breast, and liver cancers.	Hsp27 expression can increase under hypoxia and is regulated through a HIF-1α/HSF1-associated mechanism. Hsp27 promotes hypoxia tolerance, supports VEGF-associated angiogenesis, and protects cells against oxidative stress, partly through effects on antioxidant capacity and apoptosis. ROS can also regulate Hsp27 localization and phosphorylation.	[45]
Hsp10 (HspE1)	Primarily mitochondria and cytoplasm	Acts as co-chaperone of mitochondrial Hsp60. It is associated with various tumor types including prostate cancer, exocervical cancer, large bowel cancer, and serous ovarian cancer.	Hsp10 participates in the mitochondrial stress response and can protect mitochondrial function during hypoxia/reoxygenation. Experimental evidence suggests that it can reduce mitochondrial ROS and preserve metabolic reprogramming during hypoxia and oxidative stress.	[46]

## Data Availability

No new data were created or analyzed in this study. Data sharing is not applicable to this article.

## Abbreviations

The following abbreviations are used in this manuscript:

17-AAG	17-allylamino-17-demethoxygeldanamycin
17-DMAG	17-dimethylaminoethylamino-17-demethoxygeldanamycin
8-oxo-G	8-oxo-guanine
ACD	α-crystalline domain
ACAD	Acyl-CoA dehydrogenase
AhR	Aryl hydrocarbon receptor
AIF	Apoptosis-inducing factor
ALK	Anaplastic lymphoma kinase
ALL	Acute lymphoblastic leukemia
ATM	Ataxia-telangiectasia-mutated kinase
AML	Acute myeloid leukemia
APAF-1	Apoptosis protease activating factor 1
ASK1	Apoptosis-regulating kinase 1
CHIP	Hsc70-interacting protein
CAT	Catalase
CAF1	Chromatin assembly factor 1
CADD	Computer-assisted drug design
CMA	Chaperone-mediated autophagy
CML	Chronic myeloid leukemia
CRPC	Castration-resistant prostate cancer
CRC	Colorectal cancer
ER	Endoplasmic reticulum
ESCC	Esophageal squamous cell carcinoma
EPO	Erythropoietin
EMT	Epithelial–mesenchymal transition
ETC	Electron transport chain
EVs	Extracellular vesicles
FH	Fumarate hydratase
FAO	Fatty acid oxidation
GLUT1	Glucose transporter 1
GPX	Glutathione peroxidase
GR	Glucocorticoid receptor
Grp78	Glucose-regulated protein 78
GSH	Reduced glutathione
H_2_O_2_	Hydrogen peroxide
HCC	Hepatocellular carcinoma
HspA9	Mortalin
HIFs	Hypoxia-inducible factors
HMOX1	Heme oxygenase-1
HSPs	Heat shock proteins
HSFs	Heat shock transcriptional factor
HSEs	Heat shock elements
HSP90	Heat shock protein 90
Hsp70	Heat shock protein 70
Hsp60	Heat shock protein 60
HNSCC	Head and neck squamous cell carcinoma
LRP1	Lipoprotein receptor-related protein-1
MM	Multiple myeloma
MPNs	myeloproliferative neoplasms
mTOR	Mammalian target of rapamycin
NSCLC	Non-small cell lung cancer
O_2_•^−^	Superoxide
OH^−^	Hydroxyl ion
OXPHOS	Oxidative phosphorylation
PC	Prostate cancer
PKM2	Pyruvate kinase M2
PHD	Prolyl hydroxylase domain
PDH	Pyruvate dehydrogenase
P-gp	P-glycoprotein
PPP	Pentose phosphate pathway
PTC	Papillary thyroid carcinoma
PTMs	Post-translational modifications
PUFAs	Polyunsaturated fatty acids
ROS	Reactive oxygen species
RNS	Reactive nitrogen species
RT	Radiotherapy
SDH	Succinate dehydrogenase
sHsp	Small heat shock protein
SOD	Superoxide dismutase
SKCM	Skin cutaneous melanoma
TNFα	Tumor necrosis factor alpha
TRAP1/2	Tumor necrosis factor receptor-associated protein 1/2
TLR	Toll-like receptor
UPR	Unfolded protein response
VEGF-A	Vascular endothelial growth factor-A
WHO	World Health Organization
xCT	Glutamate-cystine antiporter
ZEB1	Zinc finger E-box binding homeobox 1
